# Beneficial Effects of Ginger Extract on Eye Fatigue and Shoulder Stiffness: A Randomized, Double-Blind, and Placebo-Controlled Parallel Study

**DOI:** 10.3390/nu16162715

**Published:** 2024-08-15

**Authors:** Fumiko Higashikawa, Yuta Nakaniida, Hongyang Li, Lian Liang, Keishi Kanno, Keiko Ogawa-Ochiai, Yoshiaki Kiuchi

**Affiliations:** 1 Medical Center for Translational and Clinical Research, Hiroshima University Hospital, 1-2-3 Kasumi, Minami-ku, Hiroshima 734-8551, Japan; 2 Department of Ophthalmology and Visual Science, Graduate School of Biomedical and Health Sciences, Hiroshima University, 1-2-3 Kasumi, Minami-ku, Hiroshima 734-8551, Japan; d224004@hiroshima-u.ac.jp (Y.N.); ykiuchi@hiroshima-u.ac.jp (Y.K.); 3 Department of Ophthalmology, Saneikai Tsukazaki Hospital, 68-1 Aboshi Waku, Himeji 671-1227, Japan; 4 Kampo Clinical Center, Hiroshima University Hospital, 1-2-3 Kasumi, Minami-ku, Hiroshima 734-8551, Japan; hoyalee328@yahoo.co.jp (H.L.); lianglian96@gmail.com (L.L.); ikkandoo@gmail.com (K.O.-O.); 5 Department of General Internal Medicine, Hiroshima University Hospital, 1-2-3 Kasumi, Minami-ku, Hiroshima 734-8551, Japan; kkanno@hiroshima-u.ac.jp

**Keywords:** ginger, ocular blood flow, peripheral blood flow, eye fatigue, shoulder stiffness

## Abstract

This study aimed to assess ginger extract’s impact on ocular and peripheral blood flow and its potential to alleviate eye fatigue and shoulder stiffness. This study included 100 healthy individuals aged 20–73 years with eye fatigue and shoulder stiffness. Participants were randomly assigned to receive either placebo capsules or ginger extract capsules daily for eight weeks. Ocular blood flow, peripheral blood flow, eye fatigue (visual analog scale [VAS]), shoulder stiffness (VAS), body warmth (VAS), and shoulder muscle stiffness were assessed at weeks 0, 4, and 8, respectively. No improvement in ocular blood flow was observed under the study conditions. Conversely, peripheral blood flow in deep areas was enhanced in females (*p* = 0.033). Subgroup analysis by age (≥51 or <51 years) revealed that ginger’s effect on enhancing peripheral blood flow in deep vessels was restricted in females under 51 (*p* = 0.017). Similarly, subjective complaints of eye fatigue and shoulder stiffness were improved by ginger consumption in females under 51. Body warmth was favorably changed significantly in males ≥51 years due to ginger consumption. The muscle stiffness showed no statistically significant changes. In conclusion, ginger consumption reduces eye fatigue and shoulder stiffness by enhancing peripheral blood flow in relatively young females.

## 1. Introduction

For centuries, ginger (*Zingiber officinale*) has been widely used as a spice for cooking and as a medicinal herb worldwide. The main active components of ginger are gingerols, shogaols, and zingerones, which are pungent compounds. These components show anti-inflammatory and anti-oxidative activities, as confirmed in in vitro and in vivo animal models and in cancer patients [1,2,3]. Several systematic reviews have shown that consuming ginger can alleviate nausea and vomiting during pregnancy [4,5,6,7,8,9,10] and improve metabolic syndrome [4,10,11,12,13,14,15,16,17] and pain [4,18,19,20,21,22]. Nevertheless, further research is needed to elucidate the differences among clinical trial conditions, including ginger form, dosage, consumption period, and evaluation outcomes.

Ginger is also a staple ingredient in traditional Japanese cuisine and is believed to offer various health benefits, such as improvements to thermogenetic function and blood flow acceleration. Despite the many studies concerning ginger’s effects, there is limited scientific evidence to support the claims of improved blood flow, still less in the eyes, or warming of the body, which are long-held beliefs in Japan. Consequently, additional research is necessary to explore the possibility of ginger enhancing blood flow and promoting body warmth.

In recent years, there has been a significant increase in the use of visual display terminals (VDTs), such as computers, smartphones, tablets, and video games, owing to rapid advancements in IT. Prolonged exposure to VDTs has been associated with a variety of health issues, most notably VDT Syndrome. This condition is characterized by symptoms such as dry eyes, eye fatigue, and stiffness of the neck and shoulders. The prevalence of dry eye disease in VDT users ranges from 26% to 70% [23,24]. This condition has become a pressing social issue that must be addressed because it negatively impacts an individual’s quality of life [24,25]. Studies have associated low ocular blood flow with eye diseases such as glaucoma and myopia [26,27,28]. These findings suggest that improving ocular blood flow may be an effective strategy for preventing or treating eye diseases since proper blood flow to the eye is crucial for providing sufficient oxygen and nutrients to the optic nerve.

Hence, we conducted a randomized, double-blind, placebo-controlled clinical study in individuals with symptoms similar to VDT syndrome to explore whether consuming ginger could enhance both peripheral and ocular blood flow, leading to improvements in shoulder stiffness and eye fatigue. Our evaluation not only assessed ocular and peripheral blood flow, but also recorded self-reported conditions, such as eye fatigue, shoulder stiffness, and body warmth. It also measured shoulder muscle stiffness.

## 2. Materials and Methods

### 2.1. Materials

The ginger extract powder capsule weighed 250 mg and consisted of 100 mg of ginger extract powder E (2 mg of 6-shogaol, 0.5 mg of 6-gingerol; Ikeda Tohka Industries Co., Ltd., Hiroshima, Japan), 147 mg of dextrin, and 3 mg of calcium stearate. The placebo capsule contained the same constituents as the ginger capsule, except for the ginger powder, which was substituted for 100 mg of dextrin. The capsules were provided by Ikeda Food Research Co., Ltd. (Hiroshima, Japan).

### 2.2. Subjects

Participants were recruited via advertisements. The inclusion criteria were healthy individuals between the ages of 20 and 75 who experienced both eye fatigue and shoulder stiffness. Excluded from this study were subjects with eye symptoms requiring immediate treatment, patients taking medication or dietary supplements that might impact the study results, including those intended to alleviate eye fatigue, shoulder stiffness, or affect blood flow, patients who participated in another clinical trial within the past three months, or were pregnant or nursing.

### 2.3. Study Design

This randomized, double-blind, placebo-controlled, parallel clinical study was conducted at Hiroshima University Hospital from August to December 2021. One hundred study participants meeting the inclusion and exclusion criteria determined by the principal investigator were randomly assigned to either the ginger or placebo group at a 1:1 allocation ratio. The assignment table was generated using a computer with a randomization program. The subjects were stratified by sex and allocated using the block randomization method with a block size of 4. The investigator responsible for allocation was not involved in assessing the study results. The principal investigator, physicians, and clinical staff were blinded to the randomization assignment.

The participants were instructed to take one capsule of either ginger extract or a placebo daily for 8 weeks. Clinical examinations were conducted every four weeks.

The subjects were instructed as follows: (1) to maintain their usual lifestyle, including food consumption, especially ginger in dishes, and exercise; (2) to keep a daily record of their compliance with capsule intake, health conditions, medications, and dietary supplements; (3) to record their diet, including beverages and alcohol, for three consecutive days prior to each clinical visit; (4) not to take the experimental capsules before, but rather after, the clinical examination on the day of the scheduled clinical visits; (5) not to start taking new dietary supplements; and (6) not to donate blood during the study period.

In this study, we measured the change in blood flow in the macular and optic disc areas using a Laser Speckle Flowgraphy System, LSFG-NAVI (Softcare Ltd., Fukuoka, Japan), as the primary outcome. Secondary outcomes included changes in peripheral blood flow, eye fatigue (VAS), shoulder stiffness (VAS), body warmth (VAS), and shoulder muscle stiffness. The participants’ compliance rates were evaluated based on their records. Any negative effects that occurred during the study were recorded in a questionnaire at the clinical visit, in the subjects’ diaries, and assessed using the Common Terminology Criteria for Adverse Events (CTCAE) v5.0.

This clinical trial was registered in the University Hospital Medical Information Network Clinical Trials Registry (UMIN-CTR, www.umin.ac.jp) as UMIN000044983.

### 2.4. Ocular Blood Flow

Real-time changes in blood flow were detected using LSFG-NAVI. Specifically, this system allows the calculation of ocular blood flow in a particular area of interest. This system measures blood flow using the Mean Blur Rate (MBR), a relative value and not an exact measure of blood flow speed. This study measured ocular blood flow in the macular and optic disc areas. Blood flow in the optic disc was determined by subtracting the MBR of the tissue area (MT) from that of the vascular area (MV). The average of the three measurements was used for each analysis. Data from the right eye were used for all analyses.

### 2.5. Peripheral Blood Flow

O2C (“Oxygen-to-see”, LEA Medizintechnik, Giessen, Germany) is a device that allows non-invasive measurement of tissue oxygenation and microcirculation [29,30]. The present study measured blood flow and flow velocity at depths of 2 mm (cutaneous tissue) and 8 mm (subcutaneous tissue) using O2C. The data were collected as previously described [30]. Briefly, an O2C probe was placed in the middle of the dorsal aspect of the right forearm. After the patient sat for 10 min, the microcirculation was measured continuously for 3 min. The data were obtained as an average of three minutes of measurement. The measuring room was set at a comfortable temperature.

### 2.6. Visual Analog Scales (VAS)

Visual analog scales (VAS) were used to assess the level of subjective symptoms related to eye fatigue, shoulder stiffness, and body warmth. Participants were asked to mark their symptoms on a 100 mm line, ranging from 0 to 100. The VAS indicators were as follows: for eye fatigue, 0 represented “no eye fatigue at all” and 100 represented “the most severe eye fatigue imaginable”; for shoulder stiffness, 0 represented “no shoulder stiffness at all” and 100 represented “the most extreme shoulder stiffness imaginable”; for body warmth, 0 represented “the worst feeling of coldness” and 100 represented “the best feeling of warmth”.

### 2.7. Shoulder Muscle Stiffness

The firmness of the shoulder muscles in the depression midway between the acromion and cervical spine (Jianjing, GB21) was measured using a muscle hardness meter (TDM-NA1; Sato Shouji Inc., Kanagawa, Japan).

### 2.8. Statistical Analysis

**Figure 1:**
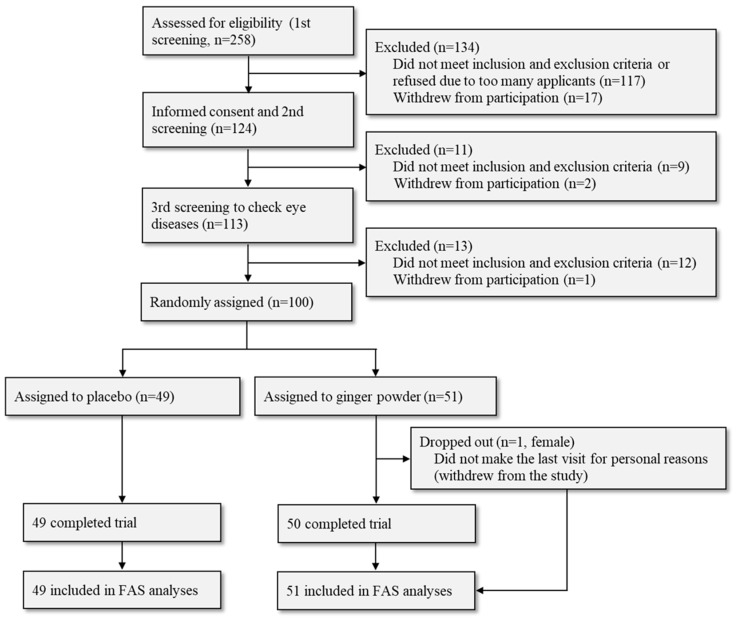
Summary of the subject flow.

We determined a sample size of 100, assuming a 10% difference between groups in outcome changes, with a standard deviation of 15%, 85% power, and 5% alpha error, because there was no reference to ginger’s effect on ocular blood flow to calculate the sample size. We assessed the data as FAS, including all cases in the final analyses, according to the intention-to-treat principle (Figure 1). The multiple imputation method was repeated 20 times to handle missing data.

We compared changes in each outcome during the 8-week intervention period between groups using Student’s t-test, except for variables that did not follow a normal distribution (shoulder stiffness (VAS), shoulder muscle stiffness, and peripheral blood flow velocity of superficial vessels), which were analyzed using the Mann–Whitney U test. For categorical data analysis, Fisher’s exact test was used to compare the groups based on safety assessment. Primary and secondary outcomes were also analyzed and stratified by sex and age (<51, ≥51 years). The age line was determined at 51 years because the average and median ages of the study participants were 51.6 and 52 years, respectively. In addition, we were interested in sex hormone effects. Data are expressed as mean ± SD or mean difference (95% confidence interval) in tables or mean ± SE in graphs. Statistical analyses were performed using IBM SPSS Statistics Version 22 (IBM, Armonk, NY, USA), with statistical significance set at *p* < 0.05.

## 3. Results

### 3.1. Population Characteristics

The flow of participants is shown in Figure 1. After screening the initial pool of applicants with an application form (first screening), 124 out of 258 participants attended the orientation session regarding the clinical trial. All 124 agreed to participate, 9 were excluded from the second screening, 2 withdrew their participation, and 113 underwent the third screening process, which included an ophthalmic examination. Finally, 100 subjects who met all the criteria were included in the study. One participant in the ginger group dropped out of the study during the last visit for personal reasons.

Table 1 summarizes the participants’ backgrounds. Choline esterase only exhibited significant differences at baseline. Table 2 shows the baseline outcomes for each group and by sex. Despite randomized allocation, there was a significant difference in the VAS for eye fatigue, with higher baseline levels in the ginger group than in the placebo group.

The subjects’ diary showed high compliance rates of 98.6 ± 2.2% in the ginger group and 98.8 ± 2.2% in the placebo group, with no notable difference between the two groups. After analyzing the subjects’ dietary records, there were no significant differences in daily calorie intake changes between the groups (46.9 ± 432.0 kcal in the ginger group and 117.7 ± 323.7 kcal in the placebo group).

### 3.2. Ginger Enhances Peripheral Blood Flow Velocity

**Figure 2:**
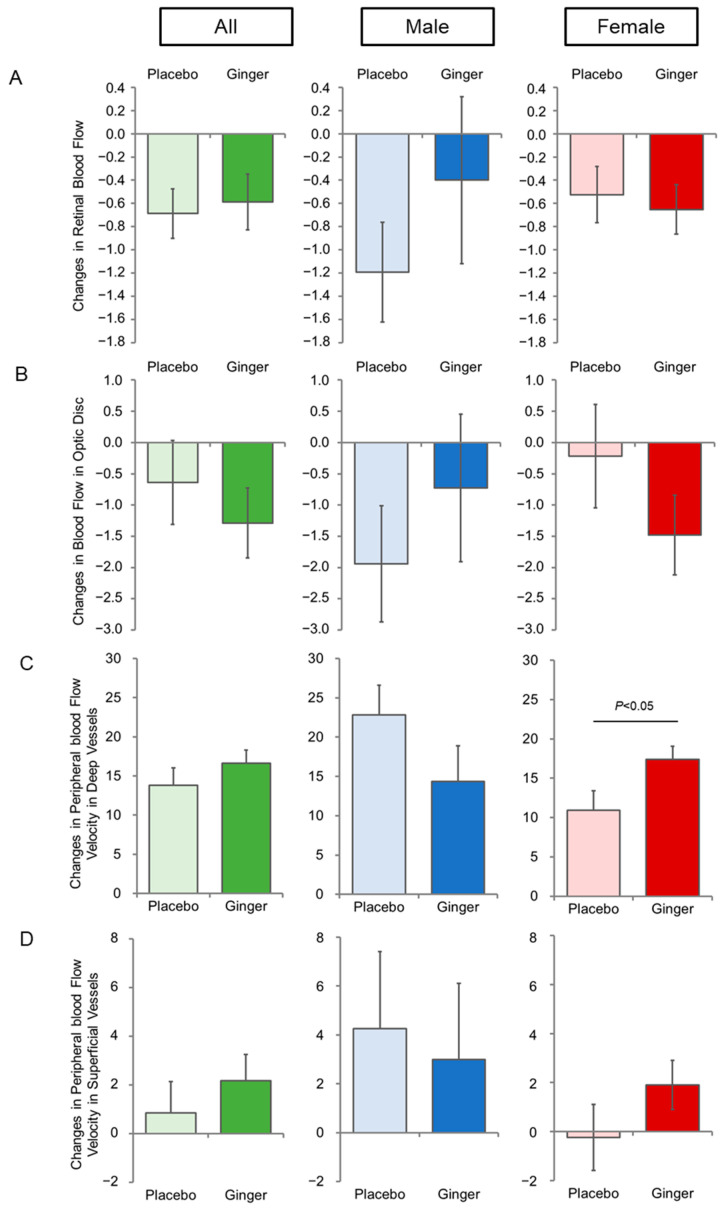
Changes in ocular and peripheral blood flow by ginger intake. (**A**) Changes in retinal blood flow; (**B**) changes in blood flow in the optic disc; (**C**) changes in the peripheral blood flow velocity of deep vessels; (**D**) changes in the peripheral blood flow velocity of superficial vessels during the 8-week intervention period. Data represent the mean ± SE.

Ocular blood flow decreased during the ginger consumption period, with no significant difference between the groups (Figure 2A,B). The tendencies did not differ between the experimental groups when subgroups according to sex were analyzed. Figure 2C shows a statistically significant increase in the peripheral blood flow velocity of deep vessels after ginger consumption only in females (*p* < 0.05). This increase was contributed by those under 51 years of age (Table 3). By contrast, there was no significant change in superficial vessel blood flow velocity among all, males, and females (Figure 2D), but only among females ≥51 years (Table 3).

### 3.3. Ginger Alleviates Eye Fatigue and Shoulder Stiffness

**Figure 3:**
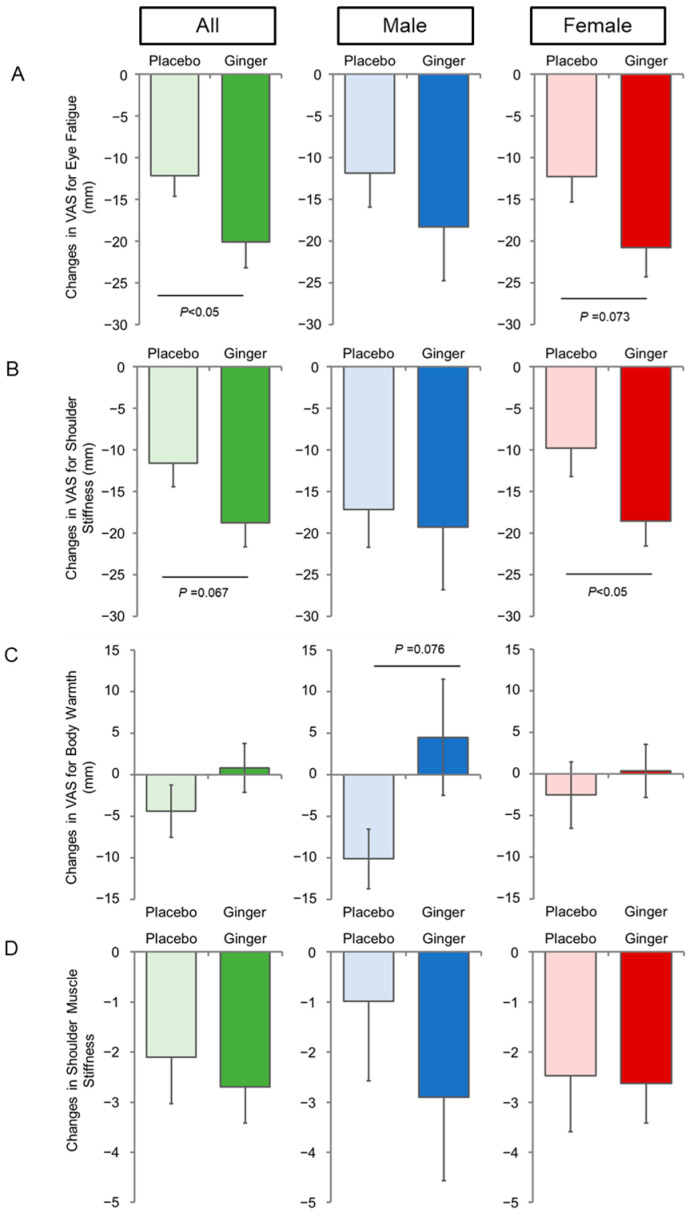
Changes in VAS and shoulder muscle stiffness by ginger intake. (**A**) Changes in VAS for eye fatigue; (**B**) changes in VAS for shoulder stiffness; (**C**) changes in VAS for body warmth; (**D**) changes in shoulder muscle stiffness during the 8-week intervention period. Data represent the mean ± SE.

Figure 3 shows the results of three types of VAS and shoulder muscle stiffness measurements using a muscle hardness meter (TDM-NA1). Self-reported eye fatigue (VAS) was reduced in both groups; however, the degree was significantly greater in the ginger group than in the placebo group (Figure 3A, “All”). Shoulder stiffness in VAS also decreased in both groups, but the ginger group decreased more than the placebo group (*p* = 0.067, Figure 3B, “All”).

Since this clinical trial was conducted from autumn to winter, self-reported body warmth was shifted toward the colder direction in the placebo group (Figure 3C). However, despite the same seasonal conditions as the parallel study, the VAS values for body warmth did not decrease from baseline in the ginger group, although there was no significant differences between the groups. 

As shown in Figure 3B, although an improvement in shoulder stiffness (VAS) was observed, there was no difference between intervention groups in the objective measurement of shoulder muscle stiffness using TDM-NA1 (Figure 3D).

Sex-dependent effects were also observed in VAS scores for eye fatigue and shoulder stiffness (Figure 3A,B). Similarly, the subgroup of younger females (<51 years) contributed to these improvements (Table 3).

### 3.4. Safety of Ginger

The amount of ginger used in this study did not exceed the range of average daily ginger consumption in Japan. Safety was assessed by the study subject’s self-reported symptoms in daily records, physical examinations, and clinical questionnaires. No safety issues related to ginger consumption were addressed (Tables S1 and S2).

## 4. Discussion

In recent years, the rapid development and diffusion of computers, smartphones, and video games have increased the prevalence of VDT syndrome, a modern malady. More office work, inadequate exercise, and VDT increase complaints of dry eyes, eye fatigue, and shoulder and neck discomfort, reducing quality of life.

We evaluated whether ginger consumption improved blood flow and/or self-reported complaints, such as eye fatigue and shoulder stiffness. Ginger accelerated peripheral blood flow; however, this effect was limited to relatively young females (under 51 years old) in stratified subgroup analyses, indicating age- and sex-dependent responses. Ginger consumption can prevent hypertension [17,18]. In a cross-sectional study examining the association between ginger intake and the incidence of various chronic diseases, subgroup analyses separated by age groups showed a negative association between increased ginger intake and the incidence of hypertension in patients aged ≥ 18 years and ≥40 years, whereas the association was lost in those aged 60 years and older [17]. A systematic review indicated that ginger’s blood pressure-lowering effects were observed only in studies with a mean age of 50 years or younger [18]. These results support the age-dependent effects of ginger on blood pressure. The mechanism of lowering blood pressure is not completely understood. However, it appears that the dilation of blood vessels may play a role based on the finding that ginger extract causes endothelium-dependent relaxation of porcine coronary arteries through the NO signaling pathway [31]. Although no change in blood pressure (systolic and diastolic) was observed during the intervention for all subgroups (stratified by sex or age) in this study, the participants were not a population with high blood pressure.

The effect of ginger on blood flow in young females implies the involvement of female hormones. Oral administration of ginger honey has been reported to induce estrogen and glutathione elevation in mice under stress conditions [32]. It has also been demonstrated that estrogen decreases cerebral vascular tone and increases cerebral blood flow by enhancing endothelial-derived NO and prostacyclin pathways, whereas testosterone has the opposite effect [33]. Furthermore, estrogen enhances NO production, drives blood vessel dilation, and increases blood flow [34,35]. Based on the evidence mentioned above, the increase in blood flow in young females in this study could be driven by blood vessel dilation from enhanced estrogen levels due to ginger consumption.

In this study, the enhancement of blood flow velocity by ginger consumption was detected mainly in the deep vessels but not superficial vessels. It is likely that superficial vessels are easily affected by the circumstances, and deep vessels more accurately reflect ginger’s effects. In addition, blood flow increased overall, regardless of the intervention group. This trend might be the result of the participants becoming more relaxed as the clinical visit progressed. However, the effect of ginger on blood flow was detected beyond the overall increase.

Similar trends were observed for self-reported eye fatigue and shoulder stiffness. In other words, females aged <51 years showed significant improvements in eye fatigue and shoulder stiffness, as observed in peripheral blood flow. It appears that these improvements result from blood flow enhancement in young females.

The feeling of body warmth increased in the ginger group as the average in “All”, “Male”, and “Female” but was not significant. On the other hand, the feeling of body warmth shifted colder in the placebo group during the intervention. Ginger’s effect on body warmth may be counteracted by changes in seasonal cold temperatures from autumn to winter. Although men under 51 years of age showed a significant increase over the placebo group in VAS for body warmth, these results must be clarified in future studies because the sample size of the subgroup was quite small.

The results were inconsistent between self-reported shoulder stiffness (VAS) and shoulder muscle hardness measured in terms of ginger effects, in which the former improved and the latter showed no significant difference. This finding may be due to the difficulty in measuring the exact same position on the shoulder during the 4-week interval measurements.

In this study, the ocular blood flow determined by LSFG-NAVI was not enhanced, but decreased in both groups, without significant differences between groups. In subgroup analyses, men under 51 years of age showed a statistical difference between the groups only in the retinal area but not in the optic area. However, similar to the results for body warmth, more studies are needed due to the small sample size. Considering that eye fatigue was significantly improved in the ginger group, it is possible that the optic nerve and surrounding tissue are delivered more oxygen and nutrients owing to improvements in ocular blood flow, thereby improving eye fatigue. This study was conducted from the beginning of autumn to winter; therefore, ocular blood flow may have been diminished by low temperatures, whereas peripheral blood flow increased under the same seasonal conditions. Reproducibility in ocular blood flow measurements in terms of variation between the measurer and different examination dates is a major concern that should be addressed in future studies.

The prevalence of myopia in middle-aged populations is relatively high in East Asia. It is more severe in preschool and school children, reflecting the rapid spread of VDT in recent years [36]. Myopia is a known risk factor for glaucoma [37]. Interestingly, the axial length in the ginger group decreased by 0.006 mm after 8 weeks of the intervention period in this study. From a clinical perspective, it seems insignificant, but if it continuously shortens, it would be −0.038 mm per year. Conversely, the placebo group showed an increase in axial length of 0.006 mm at 8 weeks, and a statistically significant difference was observed between the groups (*p* = 0.019). Moreover, the number of subjects whose axial length was reduced was significantly higher in the ginger group (17 of 51) than in the placebo group (6 of 49, *p* = 0.017 by Fisher’s exact test). According to the single-center cohort study, which investigated myopia prevalence and progression at ages 20 and 28 in Western Australia, the axial length elongated to 0.02 mm/year as an average in eight years [38]. Therefore, future clinical studies with a longer intervention period are expected to confirm the meaningful reduction in axial length by ginger consumption since it would be beneficial for myopia from a longitudinal perspective. If this is the case, ginger may potentially contribute to maintaining eye health.

The current study has three limitations. First, the number of male participants was relatively small, which could have affected the accuracy of the subgroup analysis by sex and age. Second, the degree of inter- and intra-measurer variability of ocular blood flow assessed using the LSFG-NAVI was not negligible for comparison over the intervention period. Therefore, it is possible that ocular blood flow was also increased by ginger consumption but could not be detected under the study conditions. Finally, the baseline VAS score for eye fatigue was significantly higher in the ginger group than in the placebo group. Baseline shoulder stiffness was also slightly higher in the ginger group, but the difference was not statistically significant. These were the results of randomization stratified by sex and not at baseline levels. However, the average at week 8 in both VAS scores for eye fatigue and shoulder stiffness is lower in the ginger group than in the placebo group in the “All”, “Female”, and “Female under 51 years old” subgroups. In addition, analysis of covariance (ANCOVA) for eye fatigue using the baseline value as a covariate showed significant differences between groups.

Our research was conducted using a representative Japanese population. However, future research with larger and more diverse populations, considering factors such as age, sex, ethnicity, cultural differences, socioeconomic status, and geographical location, would improve the generalizability of our findings.

## 5. Conclusions

Ginger is safe and beneficial for improving eye fatigue and shoulder stiffness by increasing blood flow velocity, especially for females under 51 years of age. These benefits are crucial to enhance quality of life since these symptoms are often understated despite being significant for the individual.

## 6. Patents

After obtaining the results of this clinical study, Hiroshima University and Ikeda Food Research Co., Ltd. jointly submitted patents for using ginger for eye fatigue and shoulder stiffness. F.H. and Y.K. are co-inventors of these patents.

## Figures and Tables

**Table 1:** Characteristics of the study participants.

	Placebo (*n* = 49)	Ginger (*n* = 51)	*p*-Value
Age (years)	51.1 ± 12.0	52.0 ± 10.2	0.694
Male/Female	12/37	13/38	-
Height (cm)	159.7 ± 7.8	159.5 ± 6.8	0.902
Body weight (kg)	55.3 ± 11.9	56.6 ± 11.6	0.626
BMI (kg/m^2^)	21.5 ± 3.3	22.1 ± 3.8	0.429
Body fat percentage (%)	24.8 ± 7.5	26.1 ± 8.1	0.482
Systolic blood pressure (mmHg)	109.9 ± 18.0	112.4 ± 15.2	0.673
Diastolic blood pressure (mmHg)	66.8 ± 12.9	68.3 ± 10.5	0.902
Heart rate (beats per min)	68.8 ± 12.5	69.7 ± 9.0	0.469
White blood cell count (×10^3^/mL)	5.23 ± 1.55	5.45 ± 1.19	0.438
Red blood cell count (×10^6^/mL)	4.57 ± 0.43	4.64 ± 0.45	0.480
Hemoglobin (g/dL)	13.6 ± 1.4	14.0 ± 1.3	0.102
Hematocrit (%)	42.3 ± 3.9	43.4 ± 3.7	0.151
Platelet count (×10^4^/mL)	24.6 ± 6.3	25.4 ± 6.0	0.532
AST (IU/L)	21.3 ± 5.0	21.0 ± 6.0	0.797
ALT (IU/L)	18.9 ± 9.2	19.6 ± 12.9	0.746
γ-GTP (IU/L)	21.4 ± 8.7	28.2 ± 33.8	0.176
LDH(IU/L)	188 ± 26	188 ± 24	0.968
Choline esterase (IU/L)	316 ± 68	345 ± 70 *	0.041
Alkaline phosphatase (IU/L)	65.9 ± 19.1	67.5 ± 16.9	0.676
Amylase (IU/L)	80.1 ± 28.9	96.9 ± 92.1	0.227
Total protein (g/dL)	7.44 ± 0.45	7.41 ± 0.37	0.708
Total bilirubin (mg/dL)	0.79 ± 0.28	0.73 ± 0.22	0.233
Albumin (g/dL)	4.57 ± 0.28	4.53 ± 0.25	0.473
Uric acid (mg/dL)	4.69 ± 1.10	4.89 ± 1.39	0.436
Blood urea nitrogen (mg/dL)	13.5 ± 3.3	13.2 ± 4.4	0.720
Creatinine (mg/dL)	0.691 ± 0.131	0.690 ± 0.165	0.968
eGFR (mL/min/1.73 m^2^)	98.9 ± 21.0	99.5 ± 21.9	0.886
Total cholesterol (mg/dL)	228 ± 42	232 ± 40	0.613
LDL cholesterol (mg/dL)	136 ± 35	141 ± 33	0.523
HDL cholesterol (mg/dL)	69.4 ± 20.4	70.8 ± 17.2	0.728
LDL/HDL ratio	2.13 ± 0.82	2.10 ± 0.65	0.846
Triglycerides (mg/dL)	110.9 ± 97.9	107.9 ± 63.6	0.856
Fasting blood glucose (mg/dL)	97.3 ± 8.7	95.5 ± 9.0	0.327

Data are expressed as mean ± SD. * Statistical difference versus placebo group by Student’s *t*-test at *p* < 0.05.

**Table 2:** The baseline values of each outcome.

	Placebo	Ginger	*p*-Value
Retinal blood flow			
All (*n* = 49, 51)	10.4 ± 3.9	9.4 ± 3.3	0.143
Male (*n* = 12, 13)	10.1 ± 3.0	9.5 ± 3.8	0.662
Female (*n* = 37, 38)	10.6 ± 4.2	9.4 ± 3.2	0.160
Blood flow in optic disc			
All (*n* = 49, 51)	34.7 ± 6.3	34.1 ± 6.3	0.588
Male (*n* = 12, 13)	30.6 ± 4.8	31.1 ± 5.8	0.819
Female (*n* = 37, 38)	36.1 ± 6.2	35.1 ± 6.2	0.480
Peripheral blood Flow Velocity in Deep Vessels			
All (*n* = 49, 51)	38.9 ± 15.1	36.6 ± 13.4	0.438
Male (*n* = 12, 13)	31.8 ± 13.5	38.0 ± 17.0	0.322
Female (*n* = 37, 38)	41.2 ± 15.1	36.2 ± 12.2	0.118
Peripheral blood Flow Velocity in Superficial Vessels			
All (*n* = 49, 51)	33.3 ± 7.7	31.5 ± 7.7	0.226
Male (*n* = 12, 13)	30.3 ± 7.8	30.6 ± 9.3	0.917
Female (*n* = 37, 38)	34.4 ± 7.5	31.8 ± 7.2	0.131
VAS of Eye Fatigue (mm)			
All (*n* = 49, 51)	57.8 ± 17.7	64.7 ± 14.4 *	0.035
Male (*n* = 12, 13)	58.9 ± 19.3	66.1 ± 16.5	0.328
Female (*n* = 37, 38)	57.4 ± 17.5	64.2 ± 13.8	0.065
VAS of Shoulder Stiffness (mm)			
All (*n* = 49, 51)	65.6 ± 20.2	68.6 ± 17.2	0.429
Male (*n* = 12, 13)	63.5 ± 24.2	67.0 ± 16.2	0.680
Female (*n* = 37, 38)	66.3 ± 19.1	69.2 ± 17.8	0.502
VAS of Body Warmth (mm)			
All (*n* = 49, 51)	52.4 ± 14.2	53 ± 16.5	0.834
Male (*n* = 12, 13)	54.5 ± 12.2	14.4 ± 4.2	0.999
Female (*n* = 37, 38)	51.7 ± 14.8	53 ± 17.4	0.740
Shoulder Muscle Stiffness			
All (*n* = 49, 51)	32.1 ± 4.8	32.4 ± 6.4	0.788
Male (*n* = 12, 13)	29.9 ± 2.6	29.1 ± 5.4	0.647
Female (*n* = 37, 38)	32.8 ± 5.1	33.5 ± 6.3	0.591

Data are expressed as mean ± SD. * Statistical difference versus placebo group by Student’s *t*-test at *p* < 0.05.

**Table 3:** Age- and sex-dependent effects of ginger. Differences during the 8-week intervention period.

	Male		Female	
	≥51 Years Old	<51 Years Old	≥51 Years Old	<51 Years Old
(*n*; Placebo, Ginger)	(*n* = 9, 9)	(*n* = 3, 4)	(*n* = 21, 20)	(*n* = 16, 18)
Retinal blood flow	−0.0 (−2.4, 2.4)	3.0 (1.5, 4.5) **	−0.2 (−1.1, 0.7)	−0.0 (−0.9, 0.8)
Blood flow in optic disc	0.7 (−3.2, 4.6)	2.4 (−1.9, 6.6)	−0.9 (−3.9, 2.1)	−1.7 (−4.5, 1.0)
Peripheral blood Flow Velocity in Deep Vessels	−12.4 (−27.9, 3.1)	2.0 (−9.9, 13.9)	2.8 (−5.2, 10.9)	10.9 (2.4, 19.5) *
Peripheral blood Flow Velocity in Superficial Vessels	−0.3 (−12.1, 11.5)	−3.1 (−12.1, 5.9)	4.3 (0.5, 8.1) *	−0.3 (−5.9, 5.4)
VAS of Eye Fatigue (mm)	−7.4 (−27.2, 12.4)	−4.6 (−31.4, 22.2)	−1.2 (−13.8, 11.3)	−16.8 (−30.9, −2.6) *
VAS of Shoulder Stiffness (mm)	−5.5 (−28.5, 17.5)	6.6 (−18.3, 31.6)	−5.8 (−19.2, 7.7)	−12.9 (−24.2, −1.7) *
VAS of Body Warmth (mm)	3.6 (−15.4, 22.5)	33.3 (9.6, 57.1) *	4.7 (−8.2, 17.7)	1.5 (−14.0, 17.0)
Shoulder Muscle Stiffness	−1.6 (−8.3, 5.1)	−3.3 (−6.7, 0.1)	0.8 (−3.4, 5.0)	−1.3 (−4.8, 2.1)

The data are the mean difference (95% confidence interval) of the ginger group versus the placebo group. * *p* < 0.05 and ** *p* < 0.01 compared to placebo group by Student’s *t*-test.

## Data Availability

The raw data supporting the conclusions of this article will be made available by the authors upon request with the right intent.

## Supplementary Materials

The following supporting information can be downloaded at: https://www.mdpi.com/article/10.3390/nu16162715/s1, Table S1: Number of subjects showing adverse events during the intervention., Table S2: Frequency of self-reported symptoms during the intervention.
