# Supplementary material for: Beneficial Effects of Ginger Extract on Eye Fatigue and Shoulder Stiffness: A Randomized, Double-Blind, and Placebo-Controlled Parallel Study
Source: Nutrients. 2024 Aug 15;16(16):2715. doi: 10.3390/nu16162715 (PMC11357383; doi:10.3390/nu16162715)
Supplement: Supplementary file 1 [file nutrients-16-02715-s001.zip › nutrients-3104228-supplementary.pdf]

Table S1 Number of subjects showing adverse events during the intervention.

	Placebo (n=49)	Ginger (n=51)	*P* value
Body weight			
Decrease, grade 1	1 (2%)	0	0.49
Increase, grade 3	1 (2%)	0	0.49
Systolic blood pressure			0.43
Grade 1	1 (2%)	2 (4%)	-
Grade 2	2 (4%)	0	-
Grade 3	1 (2%)	0	-
Diastolic blood pressure			0.67
Grade 1	1 (2%)	1 (2%)	-
Grade 2	1 (2%)	1 (2%)	-
Grade 3	1 (2%)	0	-

Data are presented as number (%). *P* values were obtained using Fisher's exact test.

Grades 1-3 were assigned according to the Common Terminology Criteria for Adverse Events version 5.0 (CTCAE v5.0).

Table S2 Frequency of self-reported symptoms during the intervention.

	Placebo (n=49)	Ginger (n=51)	*P* value
General symptoms			
Cold/Flu	1 (2%)	5 (10%)	0.20
Cough/Phlegm	2 (4%)	6 (12%)	0.27
Snivel/Sneezing/Nasal congestion	8 (16%)	8 (16%)	1.00
Throat pain	5 (10%)	6 (12%)	1.00
Fever/Chillness	3 (6%)	2 (4%)	0.67
Headache	20 (41%)	25 (49%)	0.43
Fagirue	20 (41%)	14 (27%)	0.20
Gastrointestinal disorders			
Stomachahe/Stomach upset	7 (14%)	11 (22%)	0.44
Abdominal pain/Flatulence	8 (16%)	5 (10%)	0.38
Diarrhia	7 (14%)	4 (8%)	0.35
Constipation	4 (8%)	3 (6%)	0.71
Nausea	1 (2%)	5 (10%)	0.20
Pain			
Neck, shoulder, back	37 (76%)	38 (75%)	1.00
Lower back	10 (20%)	9 (18%)	0.80
Lower limb	4 (8%)	7 (14%)	0.53
Upper limb	3 (6%)	4 (8%)	1.00
Others	7 (14%)	5 (10%)	0.55
Ocular symptom	22 (45%)	29 (57%)	0.32
(Eye fatigue, redness in the eye, bleary eyes, etc)			
Skin symptom	5 (10%)	3 (6%)	0.48
Others			
Drowsiness, agrypnia	9 (18%)	9 (18%)	1.00

Dizziness	4 (8%)	0	0.054
Stomatitis and other symptom in the mouth	10 (20%)	7 (14%)	0.43
Feeling of coldness	6 (12%)	5 (10%)	0.76
Menstrual pain	2 (4%)	4 (8%)	0.68
Psychological symptom	4 (8%)	1 (2%)	0.20
Diaphoresis	1 (2%)	2 (4%)	1.00
Nose bleeding	0	1 (2%)	1.00
Anemia	0	1 (2%)	1.00
Oral herpes infection	0	1 (2%)	1.00
Frequent micturition	1 (2%)	0	0.49
Buzzing	1 (2%)	0	0.49

Data are presented as number (%). *P* values were obtained using Fisher's exact test.
